# Supplementary material for: Iterative improvement in the automatic modular design of robot swarms
Source: PeerJ Comput Sci. 2020 Dec 7;6:e322. doi: 10.7717/peerj-cs.322 (PMC7924708; doi:10.7717/peerj-cs.322)
Supplement: Supplemental Information 3 [file peerj-cs-06-322-s003.zip › argos3/doc/api/standalone/a00614_source.html]

ARGoS: plugins/simulator/physics\_engines/dynamics2d/dynamics2d\_single\_body\_object\_model.h Source File


- Main Page
- Related Pages
- Namespaces
- Classes
- Files

- File List
- File Members

# plugins/simulator/physics\_engines/dynamics2d/dynamics2d\_single\_body\_object\_model.h

Go to the documentation of this file.

```
00001 
00007 #ifndef DYNAMICS2D_SINGLE_BODY_OBJECT_MODEL_H
00008 #define DYNAMICS2D_SINGLE_BODY_OBJECT_MODEL_H
00009 
00010 namespace argos {
00011    class CDynamics2DSingleBodyObjectModel;
00012 }
00013 
00014 #include <argos3/plugins/simulator/physics_engines/dynamics2d/dynamics2d_model.h>
00015 #include <argos3/plugins/simulator/physics_engines/dynamics2d/chipmunk-physics/include/chipmunk.h>
00016 
00017 namespace argos {
00018 
00047    class CDynamics2DSingleBodyObjectModel : public CDynamics2DModel {
00048 
00049    public:
00050 
00057       CDynamics2DSingleBodyObjectModel(CDynamics2DEngine& c_engine,
00058                                        CComposableEntity& c_entity);
00059 
00064       virtual ~CDynamics2DSingleBodyObjectModel();
00065 
00070       inline CComposableEntity& GetComposableEntity() {
00071          return m_cEntity;
00072       }
00073 
00078       inline const CComposableEntity& GetComposableEntity() const {
00079          return m_cEntity;
00080       }
00081 
00086       inline cpBody* GetBody() {
00087          return m_ptBody;
00088       }
00089 
00094       inline const cpBody* GetBody() const {
00095          return m_ptBody;
00096       }
00097 
00098       virtual void Reset();
00099 
00100       virtual void MoveTo(const CVector3& c_position,
00101                           const CQuaternion& c_orientation);
00102 
00103       virtual void CalculateBoundingBox();
00104 
00105       virtual void UpdateEntityStatus();
00106 
00107       virtual void UpdateFromEntityStatus()  = 0;
00108 
00109       virtual bool IsCollidingWithSomething() const;
00110 
00133       virtual void SetBody(cpBody* pt_body,
00134                            Real f_height);
00135 
00139       void UpdateOriginAnchor(SAnchor& s_anchor);
00140 
00141    private:
00142 
00143       CComposableEntity& m_cEntity;
00144       cpBody*            m_ptBody;
00145    };
00146 
00147 }
00148 
00149 #endif
```

---

Generated on 10 Jul 2018 for ARGoS by 
 1.6.1
